# Guillain-Barré Syndrome and Miller Fisher Syndrome in Association With an Arboviral Outbreak: A Brazilian Case Series

**DOI:** 10.3389/fmed.2022.911175

**Published:** 2022-06-28

**Authors:** Mateus Santana do Rosário, Pedro Antônio Pereira de Jesus, Daniel Santana Farias, Marco Antônio Caires Novaes, Marcos Vinicius Lima Oliveira Francisco, Cleiton Silva Santos, Daniel Moura, Fernanda Washington de M. Lima, Luiz Carlos Junior Alcantara, Isadora Cristina de Siqueira

**Affiliations:** ^1^Instituto Gonçalo Moniz, Fundação Oswaldo Cruz, Salvador, Brazil; ^2^Secretaria Estadual da Saúde da Bahia, Hospital Geral Roberto Santos, Salvador, Brazil; ^3^Hospital Santa Izabel, Santa Casa de Misericórdia da Bahia, Salvador, Brazil; ^4^Hospital São Rafael, Fundação Monte Tabor, Salvador, Brazil; ^5^Faculdade de Farmácia, Laboratório de Imunologia das Doenças Infecciosas, Universidade Federal da Bahia, Salvador, Brazil; ^6^Laboratório de Flavivirus, Instituto Oswaldo Cruz, Fundação Oswaldo Cruz, Rio de Janeiro, Brazil

**Keywords:** Guillain-Barré syndrome, Miller Fisher syndrome, Zika virus, dengue, chikungunya

## Abstract

**Introduction:**

Guillain-Barré syndrome (GBS) in association with arboviruses, such as Zika, chikungunya, and dengue, has been previously documented; however, Miller-Fisher Syndrome (MFS) and other GBS subtypes are rarely reported.

**Methods:**

We identified a series of GBS and MFS cases that were followed during the Zika virus outbreak in Salvador, Brazil (2015–2016). Blood and CSF samples were collected for virus diagnosis. In addition, serological studies to verify previous arboviral infection and electromyography (EMG) were performed.

**Results:**

Of the 14 patients enrolled, 10 were diagnosed with GBS, including three GBS subtypes (two cases of bifacial weakness with paresthesia and one case of paraparetic GBS), and four as MFS. IgM antibodies against one or more of three arboviruses were present in 11 (78.6%) patients: anti-zika IgM positivity in eight (57%), anti-Chikungunya IgM in three (21%), and anti-Dengue in one (7%) individual. A single case was positive for both anti-Dengue IgM and anti-Chikungunya IgM, suggesting co-infection. EMG revealed an AIDP pattern in all nine patients analyzed.

**Conclusion:**

The current case series contributes to our knowledge on the clinical presentation of arbovirus-associated GBS and its subtypes, including MFS, and serves as an alert to clinicians and other healthcare professionals in regions affected by arbovirus outbreaks. We highlight the importance of recognizing arboviruses in diagnosing GBS and its subtypes.

## Introduction

Guillain-Barré syndrome (GBS) is an acute, immune-mediated polyradiculoneuropathy typically occurring 2–8 weeks after viral or bacterial infection. Motor function is usually affected, beginning distally and progressing proximally over an up to 4-week period. Areflexia, sensory disturbances, and cranial nerve involvement may also occur. Diverse clinical subtypes with different neurological features have been reported, such as Miller-Fisher syndrome (MFS), Bickerstaff syndrome, and others (1).

About two-thirds of GBS patients report a prior acute infectious illness episode. Moreover, numerous infectious agents have been associated with GBS, more commonly *Campylobacter jejuni*, as well as Cytomegalovirus, Epstein-Barr virus, varicella-zoster virus, and *Mycoplasma pneumoniae* (2, 3). In addition, in regions that have experienced arboviruses outbreaks, some studies have reported GBS followed by arboviral infection, including Dengue virus (DENV), chikungunya virus (CHIKV) or Zika virus (ZIKV) (4, 5).

In Brazil, concomitant or subsequent outbreaks of these three arboviruses have imposed significant challenges to national and local health systems. DENV has circulated in the country since 1845 and is one of the main diseases that causes a relevant public health impact. Since 1981, several outbreaks have occurred (6). CHIKV, introduced in 2014, rapidly disseminated throughout the country (7). In 2015, the first cases of Zika infection were reported (8), subsequently followed by a large-scale epidemic, and clusters of newborns with congenital Zika infection resulted in the declaration of a state of public health emergency in the country.

Human ZIKV infection was considered a benign and self-limited exanthematic illness (9). However, identifying neurological complications arising from ZIKV infections, mainly related to Guillain–Barré syndrome (10) and congenital malformations (11) raised new public health concerns. The association between GBS and ZIKV infection was first reported in French Polynesia in 2013 (12) and then subsequently confirmed in a case-control study (10). In Brazil, a rising incidence of GBS has also been linked to ZIKV, mainly in the northeast region, with several cases reported in the literature (13, 14).

Chikungunya virus infection is an acute febrile illness that may cause chronic arthropathy. Neurological complications associated with CHIKV are believed to be unusual; however, reports of CHIKV-associated encephalitis increased during the 2005–2006 CHIKV outbreak on the island of La Réunion (15). Following a 2014 outbreak of CHIKV in French Polynesia, increased numbers of cases of GBS were also reported (16). Rare associations between GBS and DENV infection have been described in case reports (17). While MFS in association with arbovirus infection is rarely reported, the literature does contain some instances of cases associated with Dengue and Zika infection (18, 19).

Herein, we present a case series of patients with GBS and its subtypes, including MFS, arising from the 2015–2016 ZIKV outbreak in Salvador, Brazil. Our study aims to characterize relevant clinical and neurological features in GBS and its subtypes occurring in association with ZIKV, CHIKV, or DENV infection.

## Methods

A hospital surveillance study was performed at two reference general hospitals in Salvador, Bahia-Brazil, from May 2015 to April 2016.

### Inclusion Criteria

Patients with symptoms compatible with GBS or its subtypes were included following admission to a neurology ward at one of the participating hospitals. A single trained neurologist established a clinical diagnosis of GBS, Miller-Fisher syndrome (MFS), or other subtypes in accordance with criteria described by the GBS classification group (1).

### Exclusion Criteria

Patients or their legal guardians who did not consent to participation or patients exhibiting symptoms likely related to other plausible causes, such as cancer, bacterial infection, trauma, intoxication, metabolic disease, and other medical conditions, were excluded from the study.

### Data Collection

The collection of clinical, epidemiological, and laboratory data was performed. All participants were evaluated by a single trained neurologist (MSR) who recorded clinical data on a standardized case report form. Clinical data were recorded both during the hospital stay and after hospital discharge in an outpatient setting.

The Guillain-Barré syndrome disability scale (GBS-DS) was used to evaluate patient impairment and the severity of neurological symptoms. Scores on this scale range from 0 to6, with higher values corresponding to a greater degree of neurological dysfunction (20). The House-Brackmann scale (HBS) was also used to evaluate the severity of facial paralysis in patients affected by this manifestation, with higher values (grades 1–6) indicating greater dysfunction (21).

### Biological Samples

Serum and cerebrospinal fluid (CSF) samples were collected by the neurologist researcher for laboratory analysis; an aliquot was marked with patient identification, processed, and sent to the Gonçalo Moniz Institute (IGM-Fiocruz) for arbovirus diagnosis. All samples were conditioned and transported under refrigeration.

### Serological Diagnosis

Serological arbovirus diagnosis was performed in all samples collected for the detection of anti-DENV, anti-CHIKV and anti-ZIKV IgM antibodies by enzyme-linked immunosorbent assay (ELISA). The commercial kits Euroimmun® Dengue IgM and Chikungunya IgM (Euroimmun, Lübeck, Germany) were used for antibody detection in accordance with the manufacturer's instructions. The detection of anti-Zika IgM antibodies was performed by in-house MAC-ELISA following protocols established by the Centers for Disease and Control (CDC-Atlanta, USA) (22).

To perform differential diagnosis, serological analysis to detect toxoplasma, rubella, cytomegalovirus, Herpes, Syphilis and HIV and HTLV was performed. Indirect ELISA and/or capture ELISA were used for the detection of specific IgG and/or IgM pertaining to each infectious agent. All diagnostic tests were performed, following manufacturer protocols, on an automated or semi-automated apparatus.

### Molecular Diagnosis

Viral RNA was extracted from CSF samples using a QIAmp viral RNA mini kit (Qiagen; Hilden, Germany). Quantitative reverse transcriptase polymerase chain reaction (RT-qPCR) was performed for Zika, Dengue and Chikungunya viruses using a multiplex PCR kit (ZDC Bio-Manguinhos- Instituto de Tecnologia em Imunobiológicos, Brazil), following manufacturer protocols, on an ABI 7500 Real-time PCR system (Thermo Fisher Scientific).

### Electrodiagnostic Testing

Electromyography (EMG) and nerve conduction studies (NCS) were performed in most of the included patients. These examinations were performed by an expert neurologist trained in electrophysiology as part of patient clinical follow-up. The Nihon Kohden Neuropack® M1 MEB-9200 EMG/EP/IOM 4-channel system was used to measure electromyography and evoked potential. Electrophysiological assessments were performed using standard electromyography techniques, including motor nerve conduction studies of the median nerve (recording of the abductor pollicis brevis), the ulnar nerve (recording of the abductor digiti minimi), and the peroneal nerve (recording of the extensor digitorum brevis), as well as sensory nerve conduction studies in radial and sural nerves.

### Ethical Considerations

All participants agreed to participate in the study and signed a term of informed consent, detailing explicit information about the nature and objectives of the research undertaken in language appropriate to the educational level of the study population.

This project was submitted to and approved by the institutional review board of the Gonçalo Moniz Institute (CEP-IGM-Fiocruz; protocol no. 1184454). Risks to volunteers were considered minimal, as routine procedures were employed, i.e., the obtainment of peripheral blood or CSF collection. Refusal to participate did not affect patient treatment. Both CSF analysis and EMG examination were previously part of these patients' diagnostic routine.

### Statistical Analysis

Data entry and data management were performed using REDCap© software v.6.14.0 (Vanderbilt University, 2016). All statistical analyses were performed using the IBM-SPSS version 21 program. A descriptive analysis of the study population was initially performed. Categorical data are described as proportions with 95% confidence intervals, while numerical data are described as means ± standard deviation, or as medians with interquartile range (IQR). For all patients who underwent subsequent evaluations ~30 days after admission, mean values corresponding to GBS-DS scores and House-Brackmann (HBS) grades were calculated at each timepoint. The results obtained from these two scales were categorized to reflect the level of patient function as follows: mild (GBS-DS 0–2) or severe disability (GBS-DS 3–5); mild (HBS 1–3) or severe facial nerve palsy (HBS 4–6).

Categorical data were compared using the Chi-squared (χ^2^) or Fisher's exact tests.

## Results

During the study period, a total of 14 patients diagnosed with GBS or its subtypes were included. All but one resided in Salvador. The median age of the participants was 40.5 years (IQR: 28–52), with a slightly higher prevalence of females (57%).

Most participants (92%) reported symptoms characteristic of viral infection prior to the onset of neurological symptoms. The most prevalent symptoms were polymyalgia, skin rash, arthralgia and/or pruritus, each reported by 8 (57%) patients, while headache and/or fever were reported by 6 (42%) participants. Conjunctivitis and edema in the extremities were uncommon (7 and 14%, respectively). It is important to highlight that 8 (57%) participants reported paresthesia during the acute phase of viral infection. The median duration of viral symptoms was 4 days (IQR: 3–6.5) and the median time until onset of neurological symptoms was 10 days (IQR: 9–14) (Table 1).

**Table 1:** Demographic and clinical characteristics of 14 patients with GBS or subtypes in Salvador, Brazil.

**Characteristics**	***n* (%)**
Mean age (years)*	40.5 (28–52)
Females	8 (57)
**Acute viral symptoms**	
Polymyalgia	8 (57)
Rash	8 (57)
Arthralgia	8 (57)
Pruritus	8 (57)
Paresthesia at the onset of symptoms	8 (57)
Headache	6 (42)
Fever	6 (42)
Edema in limbs	2 (14)
Conjunctivitis	1 (7)
Duration of viral symptoms (days)*	4 (3–6.5)
Time elapsed between viral and neurological symptoms (days)*	10 (9–14)
Time elapsed between neurological symptoms and hospital admission (days)*	4 (1–10)

^*^Median (IQR 25–75%).

Ten participants were diagnosed with GBS; most (7, 70%) presented classical GBS, while three presented GBS subtypes: Two had a bifacial weakness with paresthesias and one had paraparetic GBS. In most GBS cases, symptoms compatible with acute polyneuropathy were predominant: 9 (90%) experienced paresthesia (socks and gloves pattern), muscle weakness and hyporeflexia, while 8 (80%) suffered facial paralysis. Other clinical manifestations, including dysarthria, dysphagia and ataxia were also reported (Table 2).

**Table 2:** Neurological symptoms and clinical characteristics of 14 patients with Guillain-Barré syndrome or Miller Fisher syndrome in Salvador, Brazil.

	**Guillain-Barré syndrome** **(*n*=10) *n* (%)**	**Miller Fisher syndrome (*n*=4) *n* (%)**
**Symptoms upon admission**		
Paresthesia	9 (90)	4 (100)
Sensory disturbance	9 (90)	3 (75)
Muscle weakness	9 (90)	2 (50)
Areflexia/hyporeflexia	9 (90)	1 (25)
Facial nerve palsy	8 (80)	0
Dysarthria	7 (70)	0
Inability to walk	5 (50)	0
Dysphagia	5 (50)	1 (25)
Ocular abnormality	0	1 (25)
Ataxia	3 (30)	4 (100)
**CSF fluid analysis**		
WBC/mm^3^ (mean ± SD )	10 ± 21	30 ± 42
Albumin mg/dl (mean ± SD)	117 ± 77	217 ± 284
Increased protein (>40 mg/dl)	9 (90)	3 (75)
GBS-DS (0–2)—mild disease	3 (30)	3 (75)
GBS-DS (3–5)—severe disease	7 (70)	1 (25)
HBS (1–3)—mild facial palsy	0	0
HBS (4–6)-severe facial palsy	7 (70%)	0
Treatment with IVIG	8 (80)	0
Days between admission and IVIG*	3 (1–10)	N/A
Days between neurological symptoms and IVIG*	9 (1–25)	N/A
Admission to ICU	7 (70)	3 (75)
Need for orotracheal intubation	0	0
Death	0	0
Median length of hospital stay*	9 (5–18.5)	7 (3–15.5)

^*^Median(IQR 25%-75%); GBS, Guillain-Barré syndrome; MFS, Miller Fisher syndrome; GBS-DS, Guillain-Barré syndrome disability scale; HBS, House-Brackmann scale; IVIG, intravenous immunoglobulin; ICU, intensive Care Unit; N/A, not applicable.

Four participants were diagnosed as MFS; only one presented classical MFS, while the other three presented the acute ataxic neuropathy subtype. All had symptoms of ataxia, paresthesia in the hands and feet, and sensory disturbance. A pharyngeal-cervical-brachial pattern of muscle weakness was reported in two cases. Just one out of four individuals respectively had ocular motor abnormalities, hyporeflexia or dysphagia, and none experienced facial paralysis or the inability to walk (Table 2).

Regarding serological diagnosis, IgM antibodies against one or more of the three arboviruses were detected in 11 (78.6%) participants. Eight (57%) tested positive for anti-ZIKV IgM, 1 (7.1%) for anti-DENV IgM and 3 (21.4%) for anti-CHIKV IgM. Positivity for both anti-ZIKV IgM and anti-DENV IgM was not evidenced. One case was positive for both anti-DENV IgM and anti-CHIKV IgM, suggestive of co-infection (Table 3).

**Table 3:** Neurological syndrome and arbovirus diagnosis profiles in 14 patients with GBS or its subtypes; Salvador, Brazil, 2015–2016.

**ID number**	**Neurological syndrome**	**Anti-DENV IgM**	**Anti-CHIKV IgM**	**Anti-ZIKV IgM**	**ZDC multiplex PCR**
1	Classical GBS	Negative	Negative	Positive	Negative
2	Classical GBS	Negative	Negative	Positive	Negative
3	Classical GBS	Negative	Negative	Positive	Negative
4	Classical GBS	Negative	Negative	Negative	Negative
5	Classical GBS	Negative	Negative	Positive	Negative
6	Classical GBS	Negative	Negative	Negative	Negative
7	Classical GBS	Negative	Positive	Negative	Negative
8	Bifacial weakness and paresthesia GBS	Negative	Positive	Negative	Negative
9	Bifacial weakness and paresthesia GBS	Negative	Negative	Positive	Negative
10	Paraparetic GBS	Negative	Negative	Negative	Negative
11	MFS—acute ataxic neuropathy	Negative	Negative	Positive	Negative
12	MFS—acute ataxic neuropathy	Negative	Negative	Positive	Negative
13	MFS—acute ataxic neuropathy	Negative	Negative	Positive	Negative
14	MFS—pharyngeal-cervical-brachial weakness	Positive	Positive	Negative	Negative

*GBS, Guillain-Barré syndrome; MFS, Miller-Fisher syndrome; ZDC, Zika, Dengue, and Chikungunya; ZIKV, Zika virus; CHIKV, chikungunya virus; DENV, dengue virus*.

All of the patients tested negative for HIV, HTLV, CMV, EBV, HCV, HBV, and syphilis.

Testing by multiplex qPCR for Zika, Dengue and Chikungunya in all CSF samples returned negative results for all three arboviruses.

Cerebrospinal fluid analysis revealed albuminocytological dissociation in 9 (90%) patients with GBS and 3 (75%) with MFS. Eight (80%) GBS patients were treated with intravenous immunoglobulin (IVIG) therapy, with the onset of treatment occurring on average 3 days (range: 1–10 days) after hospital admission (Table 2). Biological samples were collected prior to IVIG infusion.

Electromyography was performed in 9/14 (64%) patients (8 GBS cases and 1 MFS). Motor NCS revealed similar patterns in most cases, with prolonged distal latencies and slowed conduction, but without any reduction in the distal compound muscle action potential (CMAP). Sensory nerve action potential amplitude and sensory nerve conduction velocity were mildly altered in the radial and sural nerves, which is compatible with acute inflammatory demyelinating polyneuropathy (AIDP) with conduction block. Facial nerve demyelination was observed in 87%.

With regard to clinical severity, the majority (70%) of GBS patients presented scores between 3 and 5 on GBS-DS, denoting greater disease severity. Only one of the MFS cases presented severe disease (GBS-DS >2). In all, 10/14 (71%) were admitted to an intensive care unit, but none required mechanical ventilation support or died. The mean length of hospital stay was 11 days for patients with GBS versus 12 days for those with MFS (Table 2).

Ten patients (71%) were reevaluated 30 days after discharge (eight GBS, two MFS cases). All patients exhibited GBS-DS scores <2. Overall mean GBS-DS on admission was 2.87 compared to 0.25 upon reevaluation; a 2-point drop was observed (*P* = 0.0001). Seven of these patients presented severe dysfunction of the facial nerve upon admission (HBS > 4), yet all showed a drastic recovery when examined 30 days after discharge (HBS < 3). The mean difference between HBS on admission and reevaluation was 3.43 points (*P* < 0.001).

## Discussion

Guillain-Barré syndrome is a term used to describe a broad spectrum of acute autoimmune neuropathies. GBS presentation varies widely, as several subtypes have been identified. Some subtypes are rare, e.g., acute ptosis or acute mydriasis. Others are more common, yet usually go unrecognized by clinicians, such as paraparetic GBS, bifacial weakness with paraesthesias, pharyngeal-cervical-brachial weakness (1). MFS, an acute idiopathic polyneuritis, is characterized by ophthalmoplegia, areflexia, and ataxia. This subtype is likely a midbrain form of GBS. In contrast to other GBS case series (10), GBS subtypes were identified in half of the patients followed in this study, including paraparetic GBS and bifacial weakness with paraesthesias or acute ataxic neuropathy. Importantly, GBS subtypes may be misdiagnosed as other diseases (e.g., Bell's palsy or cerebellar disfunction) by physicians unfamiliar with the GBS clinical spectrum.

Previous infection by ZIKV was inferred by the presence of anti-ZIKV IgM antibodies in the majority of cases herein. Many studies suggest that GBS and other neurological syndromes are strongly associated with ZIKV infection (10). Even though this study was conducted during the ZIKV outbreak in our region, not all cases could be definitely attributed to ZIKV. Three (21%) patients presented positivity for anti-CHIKV IgM and one (7%) for anti-Dengue IgM. This finding was expected, since the other co-circulating arboviruses (CHIKV and DENV) in this area have also been linked to neurological disorders, including GBS (23).

One case of MFS was positive for both anti-CHIKV IgM and anti-DENV IgM, suggesting the occurrence of co-infection. Arboviral co-infection has been reported in acute neurological syndromes and could be responsible for atypical clinical presentations (24).

Importantly, none of the patients tested positive for both anti ZIKV IgM and anti-DENV IgM antibodies, which indicates that anti-ZIKV IgM positivity did occur due to cross-reactivity. In all 11 cases presenting IgM antibodies against arboviruses, diagnosis can only be inferred as all individuals were negative under molecular testing by Multiplex qPCR and viral isolation was not performed. However, since GBS is an immune-mediated syndrome and the onset of neurological symptoms typically occurs days to weeks after infection, the etiological diagnosis of GBS is mostly based on serological testing, and few studies have described positivity for DENV, CHIKV or ZIKV by PCR (10).

Cerebrospinal fluid analysis demonstrated the presence of albuminocytological dissociation in 90% of the GBS patients and 75% of MFS cases, which is similar to two other case-control studies previously published in French Polynesia and Colombia (10, 25).

Prolonged distal latencies and reduced distal CMAP on EMG studies can be interpreted as facial nerve demyelination and conduction slowing and blockage, leading to the classification of patients as AIDP with axonal degeneration, which is consistent with a pattern of GBS. Most of our patients (87%) presented demyelinating polyneuropathy and secondary axonal degeneration of the facial nerves. The single MFS patient submitted to EMG also presented a pattern consistent with AIDP in the absence of facial nerve dysfunction. The pattern of demyelination found in the present study is similar to that seen in a case-control study conducted in Colombia (25), yet differs from another case-control study in French-Polynesia that observed an axonal polyneuropathy pattern on EMG (10).

GBS-DS and HBS evaluations are rarely reported in other GBS cohorts associated with arbovirus infection (10, 25). Most (70%) of the GBS patients in this study presented with GBS-DS scores of 3 or higher on admission, denoting severe disease. This was also reflected by a high frequency of facial nerve palsy (80%) and HBS scores >4 (88%). Patients with MFS presented with milder disease, with a predominance of GBS-DS scores <3 (75%). As expected, none of the MFS patients had facial nerve palsy. Despite severe GBS-DS presentation upon hospitalization and a high frequency of ICU admission in these patients, all cases recovered.

All patients re-evaluated 30 days after hospital discharge presented markedly less severe neurological symptoms (GBS-DS <2 and HBS <3). This stands in contrast to other GBS studies indicating that patients with higher scores on these scales tend to exhibit low rates of recovery (26). We hypothesize that the favorable rates of recovery seen herein may be due to prompt treatment with IVIG, which was administered at a median time of 3 days following admission. It is also possible that this was due to the result of GBS syndrome in association with arbovirus (27).

The clinical picture of ZIKV-associated GBS, as well as GBS occurring in association with other arboviruses, is generally similar to GBS arising from other causes. However, a recently published review indicated that cranial nerve palsy and autonomic dysfunction are more frequently associated with GBS following arbovirus infection, and that the clinical course of the disease is shorter, with a brief plateau phase and a high proportion of facial nerve involvement (23). Nonetheless, more consistent studies need to be conducted to define whether clinical peculiarities are related to arbovirus-associated GBS. Herein, we found a high proportion of GBS subtypes and facial nerve palsy, with EMG findings compatible with AIDP. In addition, all patients had favorable outcomes, with clinical improvement noted after 30 days of reevaluation.

The concept that Classical GBS could be associated with emergent arboviruses has been well-established. However, the diagnosis of MFS and other GBS subtypes still poses significant challenges, consequently leading to misdiagnosis or the underreporting of GBS cases. Our case series offers additional information on clinical presentation and follow-up of arbovirus-associated GBS and its subtypes and serves as an alert to clinicians and other healthcare professionals in regions affected by arbovirus outbreaks. Furthermore, recognizing GBS and its subtypes is essential to providing prompt treatment and supportive care for patients to prevent mortality and long-term sequelae. Accordingly, we would like to emphasize the need to carry out the diagnosis for arboviruses in patients with GBS and its subtypes, including MFS.

## Data Availability Statement

The raw data supporting the conclusions of this article will be made available by the authors, without undue reservation.

## Ethics Statement

The studies involving human participants were reviewed and approved by Comitê de Etica e Pesquisas do Centro de Pesquisas Gonçalo Moniz- Fiocruz. The patients/participants provided their written informed consent to participate in this study.

## Author Contributions

IS and LA contributed to the study design. MR and IS contributed to data analysis and writing of the manuscript. MR, PJ, DF, and MN contributed to participant enrolment, review of medical records, and the collection of samples and data. MF, CS, DM, and FL contributed to laboratory analysis. All authors contributed to the article and approved the submitted version.

## Funding

This work was supported by MCTI-Ministry of Science, Technology, Innovation/FINEP–Funding Authority for Studies and Projects/FNDCT–National Fund for the Development of Science and Technology (04160060-00/2016), and the Excellence in Research Program (PROEP) of the Gonçalo Moniz Institute (IGM-Fiocruz) (077/2020).

## Conflict of Interest

The authors declare that the research was conducted in the absence of any commercial or financial relationships that could be construed as a potential conflict of interest.

## Publisher's Note

All claims expressed in this article are solely those of the authors and do not necessarily represent those of their affiliated organizations, or those of the publisher, the editors and the reviewers. Any product that may be evaluated in this article, or claim that may be made by its manufacturer, is not guaranteed or endorsed by the publisher.
